# Development of ‘learn to dare!’: An online assessment and intervention platform for anxious children

**DOI:** 10.1186/s12888-020-2462-3

**Published:** 2020-02-11

**Authors:** Ellin Simon, Eva de Hullu, Susan Bögels, Peter Verboon, Petra Butler, Wendy van Groeninge, Wim Slot, Michelle Craske, Stephen Whiteside, Jacques van Lankveld

**Affiliations:** 1 Open University of the Netherlands, PO box 2960-NL, 6401 DL Heerlen, Netherlands; 2 Amsterdam University, Nieuwe Achtergracht 127, PO box 15804, 1001 Amsterdam, NH Netherlands; 3 UCLA, 3229 Franz Hall, Mail Code 156304, Los Angeles, CA 90095 USA; 4 Mayo Clinic, 201 W Center St, Rochester, MN 55902 USA

**Keywords:** Child anxiety, Online intervention, Early intervention, Inhibitory learning, CBT

## Abstract

**Background:**

Many children and adolescents suffer from problematic levels of anxiety, but the multitude of these children do not receive an intervention. It is of importance to increase the accessibility and availability of child anxiety interventions, as to identify and treat anxious children early and successfully. Online platforms that include information, assessments and intervention can contribute to this goal. Interventions for child anxiety are frequently based on Cognitive Behavioral Therapy, because of its strong theoretical and empirical basis. However, the working mechanisms of Cognitive Behavioral Therapy in children are poorly studied. To our knowledge, mediation studies on child anxiety are non-existent regarding online Cognitive Behavioral Therapy.

**Methods:**

We will aim at children aged 8–13 years with problematic anxiety. We recruit these children via the community setting, and refer them to our online platform ‘Learn to Dare!’ (in Dutch: ‘Leer te Durven!’), https://leertedurven.ou.nl, where information about child anxiety and our research is freely accessible. After an active informed consent procedure, the participants can access the screening procedure, which will select the children with problematic anxiety levels. Thereafter, these children will be randomized to an online intervention based on Cognitive Behavioral Therapy (*n* = 120) or to a waitlist control (WL, *n* = 120). The intervention consists of 8 sessions with minimal therapist support and contains psycho-education, exposure (based on inhibitory learning), cognitive restructuring and relapse prevention. Child anxiety symptoms and diagnoses, cognitions, avoidance behavior and level of abstract reasoning are measured. Assessments are the same for both groups and are performed before and after the proposed working mechanisms are offered during the intervention. A follow-up assessment takes place 3 months after the final session, after which children in the waitlist control group are offered to take part in the intervention.

**Discussion:**

This protocol paper describes the development of the online platform ‘Learn to Dare!’, which includes information about child anxiety, the screening procedure, anxiety assessments, and the online intervention. We describe the development of the online intervention. Offering easy accessible interventions and providing insight into the working mechanisms of Cognitive Behavioral Therapy contributes to optimizing Cognitive Behavioral Therapy for anxious youth.

## Background

Anxiety is a normal and functionally relevant psychological phenomenon. However, many children and adolescents suffer from problematic levels of anxiety. Anxiety disorders are the most prevalent psychiatric disorders in youth. Methodologically strong studies show that the prevalence of anxiety disorders in children under the age of 12 years is around 5 % (see review, [1]). A meta-analysis on the age of onset of mental disorders found that separation anxiety disorder, specific phobia and social anxiety disorder most commonly start before the age of 15 [2]. Early anxiety symptoms often develop into full anxiety disorders, and anxiety disorders are unlikely to remit when left untreated [3]. Anxiety disorders negatively affect children’s quality of life and incur high societal costs [4, 5]. Furthermore, anxiety disorders frequently precede other psychiatric disorders, such as depression and substance abuse [6]. For all these reasons, it is important to identify and treat anxious children early and successfully.

### Barriers for anxious children to access help for anxiety difficulties

As much as 70 to 80% of the children with anxiety disorders does not receive treatment [7, 8]. This high percentage is strongly determined by the low accessibility and availability of anxiety treatments, which is, to a large extent, due to the limited number of qualified or specialized therapists [9], to the stigma associated with finding psychological help, and by geographical limitations (P. C [10]). It is, therefore, of importance to increase the accessibility and availability of child anxiety interventions.

### Online CBT-based child anxiety interventions

A way to overcome the low accessibility and availability of child anxiety interventions is by providing online information, online assessments and offering online Cognitive Behavioral Therapy (CBT). The effect of face-to-face CBT appears to be comparable to the effect of online CBT, according to Vigerland and colleagues [11] and Ebert et al. [12]. Online intervention are stand-alone to a high degree, as therapists have a limited role [13]. Due to its highly structured character, CBT is suitable for conversion to online intervention programs [14].

We searched literature of published randomized controlled trials (RCT’s) on online CBT interventions and treatments for child anxiety difficulties. We excluded studies on CD-ROM based or computerized interventions and retrieved eight RCT’s in total. Of interest, not one of the eight RCT’s on online CBT in anxious children explicitly studied mediators (mechanisms of change). Moreover, moderators were only occasionally taken into account. It, thus remains uncertain how and for whom online CBT(−based interventions) for child anxiety are effective. Of these eight RCT, four focused on adolescents [15–18], one on the parents of preschoolers [19] and three target primary school children [20–22]. All three of the studies that targeted primary school children showed significant better improvement of child anxiety in the treatment condition than in the control group. We also aim at intervening via primary school children. However, in contrast to these earlier three RCT’s we do not rely strongly on active parental involvement, but, rather, focus on intervening via the children themselves.

### Exposure and cognitive restructuring

Approximately 65% of children and adolescents with anxiety disorders are free of their primary anxiety disorder after CBT treatment (James, Soler, & Weatherall [23];). Although this is a high percentage, it also shows that about one third of the anxious children does not benefit enough from CBT. If the working mechanisms of child CBT are understood, then scientists and clinicians can adapt and improve its content for children [24].

According to CBT’s theoretical model, cognitive processes determine behavior and feelings. Cognitive processes, such as thoughts, determine how someone reacts behaviorally and emotionally. In CBT, it is assumed that, through practice, one can become aware of and change dysfunctional cognitions. In turn, one can adapt his/her behavioral and emotional reactions to events (D [25].; K [26].). Anxious persons tend to overestimate the likelihood of threatening situations, while they underestimate their own cognitive and behavioral coping strategies in threatening situations [27]. Feelings of anxiety are commonly stronger or longer-lasting than necessary and anxious children often believe they cannot adequately handle issues that scare them.

Avoidance is the most important behavioral coping strategy of anxious children (P. M [28, 29].). By avoiding, the child can escape what he/she perceives as a scary situation. If a child avoids, he/she cannot learn to handle anxious feelings nor situations that frighten him/her. Additionally, the child cannot recive information that could disconfirm is/her fearful cognitions (P. C [30, 31]). By responding in an avoidant manner to feelings of anxiety, the anxiety is maintained and can even worsen.

In order to break the vicious circle of cognitions, avoidance and feelings of anxiety, CBT and CBT-based interventions mainly focus on reducing dysfunctional cognitions (e.g. the overestimation of danger) and avoidance. Dysfunctional cognitions are targeted by cognitive restructuring. During cognitive restructuring, a child learns to detect his/her dysfunctional cognition and to replace these by a functional one. Avoidance is targeted using exposure exercises. During exposure exercises, a child exposes him−/herself to anxiety inducing stimuli.

CBT thus has a strong theoretical basis that is supported by empirical studies, convincingly demonstrating that CBT is currently an effective treatment for anxiety symptoms and anxiety disorders. To date, it is unknown whether CBT for children with anxiety actually works via decreasing dysfunctional cognitions and via decreasing avoidance. Studies on CBT’s working mechanisms in children are sparse. One of the most important prerequisites of examining working mechanisms is to establish that the outcome only changes after the active ingredients considered part of the working mechanisms has been offered [32]. This prerequisite is seldomly complied with. To our knowledge, mediation studies on child anxiety CBT are non-existent regarding online CBT and only a few mediation studies exist in which CBT was performed in a face-to-face format ([33]; P. C [34–37]). Of these face-to-face studies, only Hoogendoorn’s study (2014) meets the prerequisites of testing mediation [32]. Unfortunately, avoidance behavior was not taken into consideration in Hoogendoorn’s study. Therefore, we cannot draw firm conclusions on CBT’s effect on child anxiety via alteration of cognitions and avoidance behavior, and we cannot draw any conclusions about CBT’s working mechanisms when offered in an online format.

### Inhibitory learning

Exposure is viewed as a fundamental part of CBT for anxiety disorders [38]. To date, exposure therapy in children has been based on the habituation model. This model poses that success in therapy follows from reductions in expressed fear throughout exposure trials. Fear is, therefore, monitored during exposures and is seen as an indicator of successful exposure. Most commonly, a hierarchical exposure is applied for this purpose in children: children rank their feared situations and exposure takes place in stepwise (predictable) order, least feared situation first, most feared situation last. However, although exposure reduces anxiety, there is no direct evidence that this works through the mechanism proposed in the habituation model [39]. In fact, fear reduction has been found to be non-predictive of subsequent anxiety [38].

Instead, inhibitory learning has been forwarded as a promising venue [38, 40, 41]. To grasp inhibitory learning, one must understand Pavlovian fear conditioning. When a neutral stimulus (conditional stimulus: CS) is repeatedly followed by an aversive stimulus (unconditional stimulus: US), in time a CS-US association is formed and the neutral stimulus will evoke an anticipatory fear reaction (conditional reaction: CR). This conditioned fear reaction can be decreased powerfully by extinction procedures, when the conditioned stimulus is repeatedly offered without the aversive stimulus (i.e. CS without US). Exposure is viewed as the clinical analogue of extinction, and inhibitory learning as extinction’s most important working mechanism. In contrast to habituation-based exposure, inhibitory learning does not specifically aim to reduce the anxiety levels of the person undergoing exposure. In fact, anxiety levels usually increase while applying inhibitory learning-based exposure. According to inhibitory learning models, an additional inhibitory association (CS – no US) is acquired in addition to the original CS-US association. Put simply, new information is learned that can override the old information. The extent to which new information can overrule the old information depends on the strength of the learning experience. Children who are at risk for developing anxiety disorders have deficits in inhibitory learning [40]. These deficits may hinder them from optimally profiting from exposure interventions. It is, therefore, important to create optimal inhibitory learning opportunities for anxious children in interventions. Strong learning experiences, in which the expectancy of the child about the feared situation is violated clearly and explicitly are seen as optimal inhibitory learning opportunities. This is why exposure based on inhibitory learning leans strongly on variability, and not on predictable steps of increasingly difficult exposure to the feared stimuli. During exposure based on inhibitory learning, variable stimuli, variable durations, variable levels of intensity of the exercise and variable levels of anxiety provocation are applied. Some child intervention studies for anxiety related difficulties already paid attention to making the child’s expectations about the feared situation explicit and monitoring and/or evaluating the child’s expectation during and after exposure ([42]; Philip C [43–47]). However, to our knowledge, no study exists that specifically applied variability in their exposure to create optimal inhibitory learning opportunities.

### Children’s cognitive development

CBT was originally developed for adults. Children and adolescents have unique developmental needs that should be addressed in treatment protocols and online interventions (Paula Maria [48, 49]). In their meta-analysis, Durlak, Fuhrman & Lampman [50] concluded that the effect size of child CBT was almost half the size in 5–11 year old children compared to 11–13 year olds. In line, Grave and Blissett [51] argue that the cognitive demands of CBT suit the cognitive developmental level of children aged 11 year and older better than the cognitive developmental level of younger children. In order to test whether CBT offered to primary school children is developmentally suitable, it is thus of importance to include the child’s cognitive developmental level as a potential moderator.

### Learn to dare study

**Fig. 1:**
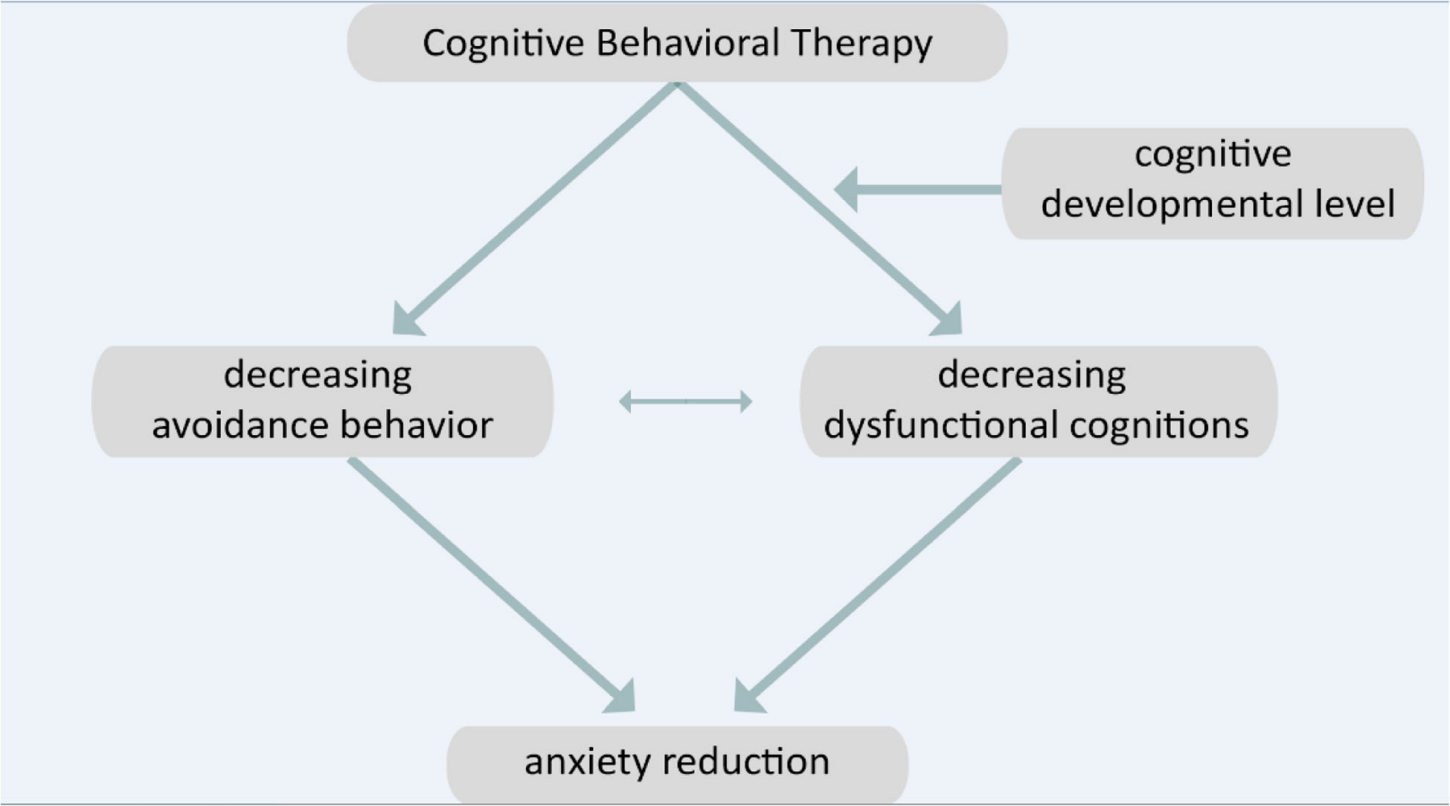
‘Learn to Dare!’ research model

The current study has five aims. First, we examine the efficacy of ‘Learn to dare!’, an online CBT program that targets anxiety without parental support and minimal therapist support. We expect that the intervention ‘Learn to Dare!’ will decrease the anxiety levels of children stronger than the anxiety levels of children in the waitlist control group. Second, we study the efficacy of exposure based on the inhibitory learning model. It is expected that exposure based on inhibitory learning will decrease child anxiety levels than the anxiety levels of children in the waitlist control group. Third, we examine whether decreases in dysfunctional cognitions and avoidance behavior are CBT’s working mechanisms. We expect that a decrease in child CBT will be mediated by decreases in dysfunctional cognitions and avoidance behavior. Fourth, the relative contribution of both proposed working mechanisms is explored. Fifth, we study whether the child’s cognitive developmental level differentially affects the proposed working mechanisms. We expect that the child’s cognitive developmental level directly affects the decrease of dysfunctional cognitions, while it does not directly affect the decrease of avoidance. In case of a high cognitive developmental level, a synergistic positive effect of the working mechanisms is expected to take place (see Fig. 1). The study design is a randomized two-group factorial design with repeated measures of the dependent variables. Intervention versus control group makes the between subjects factor and time is the only within subjects factor.

## Methods

### Learn to dare! Platform

Information for the participants, the screening procedure, the interventions and most assessments all take place on the ‘Learn to Dare!’ (In Dutch: ‘Leer te Durven!’) platform, https://leertedurven.ou.nl. Drupal version 7.x was our development environment, which is an Open Source Content Management System (CMS). Drupal is a web-based environment that thus runs in a browser on personal computers, and on handheld devices such as tablets and smartphones. The choice for developing in Drupal was made because of its globally organized community of developers and users and variety of well-maintained modules. On the server-side, Drupal requires Linux Apache MySql PHP (LAMP), see https://www.drupal.org/docs/7/system-requirements for more technical requirements. All missing functionality is self-written in three modules, extendable on Drupal 7 in PHP.

We have taken care of secure transfer of information, by running the ‘Learn to Dare!’ platform under the https protocol and using a separate server for the user-information database that is only accessible through our own local network by a dedicated server. Privacy is GDPD-compliant, for example, notification e-mails sent out to participants do not contain names and personal content.

### Participants

We aim at recruiting children aged 8–13 years with high levels of anxiety, specific phobias or other mild anxiety disorders that are included in the fifth edition of the Diagnostic and Statistical Manual of Mental Disorders (DSM-5): social anxiety disorder, separation anxiety disorder, generalized anxiety disorder, panic disorder, selective mutism, agoraphobia. With mild we mean that the disorder does not strongly interfere with daily functioning, or, in other words, does not have a high severity. By performing a simulation study with 500 replications [52] with the package “lme4” [53] in R (R Core Team, 2013), we calculated how many children from this population should be included in our sample. For this simulation we assumed a medium direct effect of the training, a small effect of the indirect pathways via the mediators and 10% random drop-out between measurements. To assess the effect of the intervention, we include a waitlist control group in addition to the intervention group. In order to obtain 80% power of the direct and indirect pathways, we need a total sample size of 240 (120 children in the intervention group and 120 children in the waitlist control group). In principle, the inclusion of participants will continue until we reach this number. We exclude children with severe anxiety diagnoses, as determined by a trained clinician during a diagnostic interview. No further exclusion criteria are applied. We will recruit our participants via the community (i.e., non-clinical/ non-refferred) in the Netherlands, a list of study sites can be obtained via the first author.

### Procedure

**Fig. 2:**
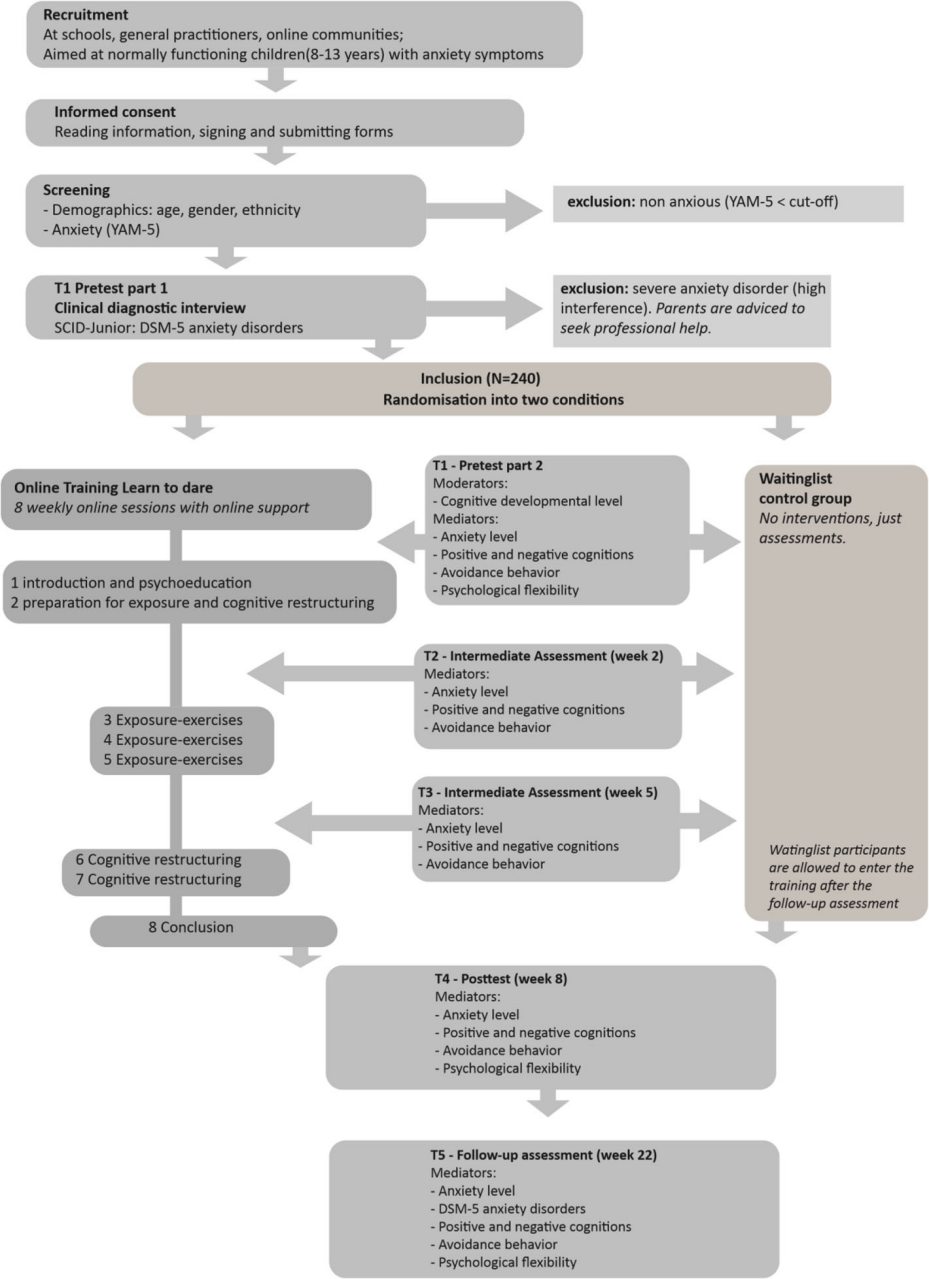
Flow diagram of ‘Learn to Dare!’ study

This study was ethically approved by a national medical ethical review board. If important changes are made to the study protocol, the medical ethical review board will be informed about this. All data and materials of this study will be publicly accessible at osf.io/d8c4p. A detailed study design and analysis plan were preregistered at https://osf.io/g2avh. The children are recruited via online advertisements, via press releases and interviews, via social media, via free publicity in magazines and papers, via flyers we hand out at primary schools, via general practitioners and preventive mental health centers. In the online information and flyers, we refer participants to the online ‘Learn to Dare!’ platform. When visiting our platform, children and their parents find information about child anxiety and the ‘Learn to Dare!’ project. They can download separate information for young children (below 12 years) and older children (12–13 years old). See Fig. 2 for the flow diagram of this study.

Parents (and children, when they are 12 or older) send a signed informed consent form to our university, after which they receive an automatically generated e-mail with their account information. With this account, the children have access to the screening questionnaire on the platform. The authors that will be responsible for data analyses do not have access to the accounts and are completely blinded from participants’ information.

We use the Youth Anxiety Measure for DSM-5 (YAM-5, [54, 55]) as a screening tool and to measure the development of anxiety over time. When completing the YAM-5, children also fill in their demographic features. Children who score in the highest quartile are labelled as high-anxious. We use different cut-offs for boys and girls in order to increase the chance of a proportional influx of boys and girls. All other children are excluded and receive a message that they are not severely anxious, and that the program is not intended to suit their level of anxiety. These children receive links to general anxiety websites for children.

Consecutively, the high-anxious children are invited by e-mail to book a date for a diagnostic interview. The diagnostic interview Structured Clinical Interview for DSM-5 Disorders for Children (SCID, [56]) is performed by telephone with a trained interviewer. If the SCID shows that a child suffers from a severe anxiety disorder other than specific phobias, the child is excluded from the study. In this case, the first author does get access to the accounts of the participants (unblinding) as the parents are contacted by phone. Parents are advised by the first author to seek help for their child at a local mental health care institution.

The study concerns a randomized controlled trial (RCT). Within the ‘Learn to Dare!’ platform, the included high-anxious children are automatically randomized to the intervention group or to the waitlist control group. Children in the waitlist control group can participate in the intervention too after the last assessment point. We apply block randomization with child’s age and sex as strata. If children are randomized to the intervention group, they are assigned personal trainer who helps them during the training. The intervention and assessments are aimed at the children only.

Children in both groups perform the same assessments, which are all online within the ‘Learn to Dare!’ platform. Assessment points are aligned with the mediators, which means they take place before and after exposure and cognitive restructuring is offered to the children. At every assessment point child anxiety, avoidance behavior and dysfunctional cognitions are measured. The assessments are embedded in the intervention program, and take place after session 1 (after psycho-education and before the child’s expectancies are made explicit), after session 3 (before the start of actual exposures), after session 5 (before the start of cognitive restructuring), and at the end of the training. Control children receive automatically generated e-mails to notify them they have to complete an assessment. A follow-up assessment takes place 12 weeks after the last session. Both the intervention children and control children receive an automatically generated e-mail to notify they have to complete this assessment. At the follow-up assessment, the children take part in the diagnostic interview again. If children do not complete an assessment or intervention session, they will receive automatically generated reminders by e-mail twice. A research assistant also monitors the progress of the children and of the interviewers and trainers.

Conform ethical procedure, in case children or their parents do not want to participate in the study at any stage for any reason, they can end their participation without having to provide a reason for this. We stimulate adherence by personal e-mails or telephone calls. The research assistant ask the participants who want to discontinue the intervention, if the participant is still willing to complete the assessments, but does not enforce this on the participants. There are no criteria, other than the participant’s wishes, for discontinuation of the intervention. Under no circumstance can children participate in a different group than the one they were randomized to. Children are free to follow a different start of treatment. Those who do during the research period, are asked to report this to the research team, so we can take this into account when performing the analyses. The ethical committee decided that there is no need to compose a data monitoring committee for this study. In case a participant is harmed during the study, the first author will be unblinded from the participant’s information and will notify the ethical committee about the harmed participant.

### Intervention ‘Learn to Dare!’

The intervention ‘Learn to Dare’ is based on Cognitive Behavioral Therapy. The intervention focuses on the anxious children themselves and are provided on an individual basis. Parents are informed about the intervention’s content, and are asked to motivate the child to progress through the training. Children and parents also have to agree on the rewards children receive after the exposures. Parents do not function as layman-therapists. Every child is assisted through the online program by a personal trainer, who holds a bachelor in psychology. The trainers work via a detailed protocol and write personalized messages to guide the child via private messaging (PM). Adherence is stimulated by the parents’ and trainer’s encouragement. Trainers are instructed and supervised by the first author.

**Fig. 3:**
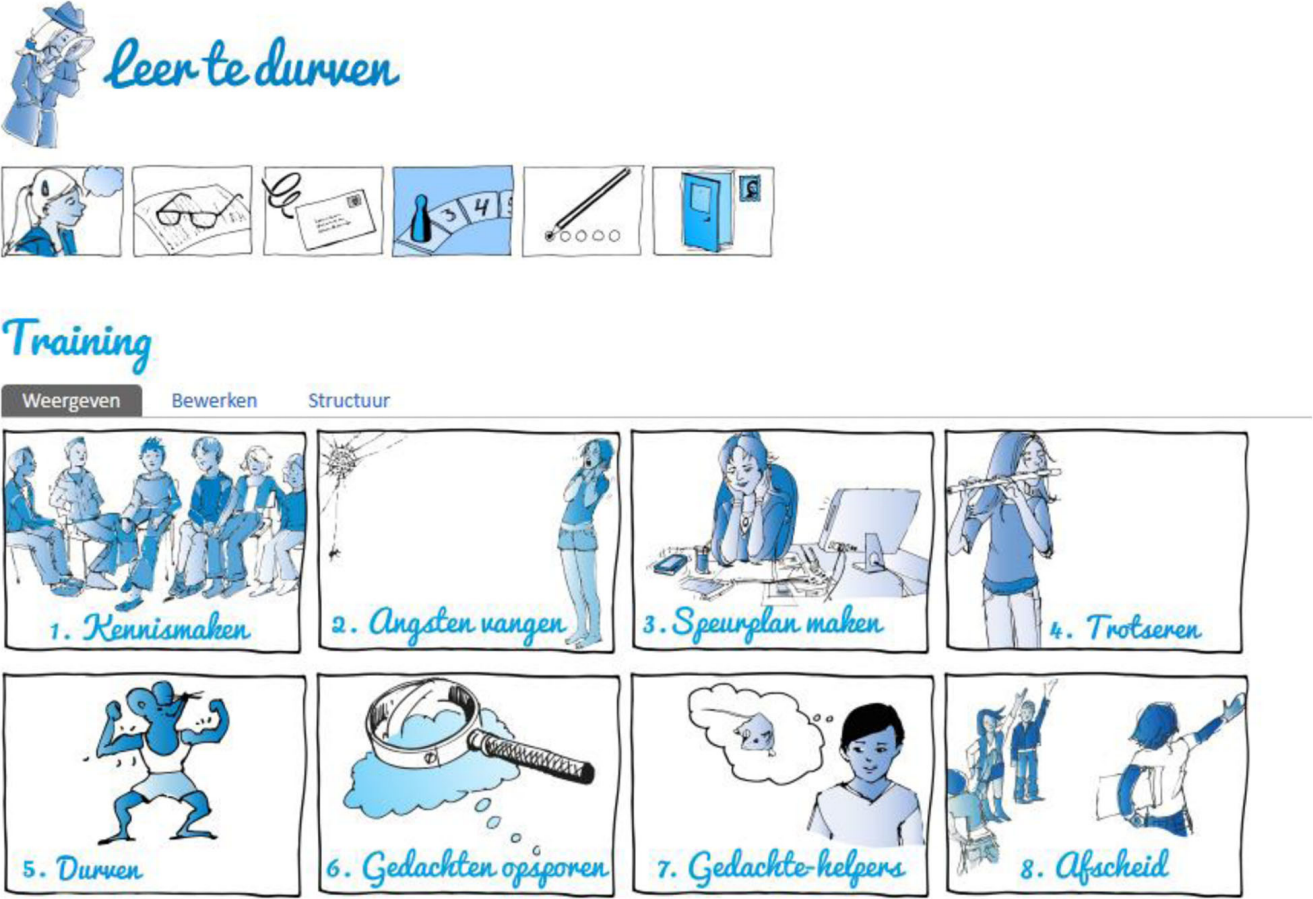
‘Learn to Dare’ intervention. *Note.* Sessions in consecutive order: 1. Getting to know each other, 2. Catching anxieties, 3. Making tracking plans, 4. Defy, 5. Dare, 6. Tracing thoughts, 7. Thought helpers, 8. Saying goodbye; Source of images: Simon, E., & Bögels, S. M. (2013). *Leer te durven! Werkboek*. Houten, Lannoo Campus. The first author has obtained written permission to use and adapt the figures for research purposes

The intervention takes place on the ‘Learn to Dare!’ platform, where the child has his/her own online workbook (see Fig. 3), the child is encouraged to act like a detective, studying his/her own anxieties. The detective theme is apparent throughout the intervention. The trainer has access to the child’s workbook and controls whether children can progress or not with the training by releasing new parts of the intervention. The trainer also enters information in an automated progress sheet that functions on the background of the workbook and automatically inserts individualized texts in the child’s workbook. Neither interviewers nor other researchers have access to the child’s workbook.

At the start of the intervention, the trainer has to select two domains of anxiety that are targeted in the Learn to Dare program. These anxiety domains are selected by the following method: First, the trainer checks the SCID-Junior data to see if a DSM-5 classification exists for this child. If one or two classifications apply, these are selected to be targeted in the intervention (e.g. fear of heights; social anxiety). In case of more than two classifications, specific phobias are given priority over other anxiety disorders. In case of no DSM-5 classifications, the trainer checks the YAM-5 data and picks two types of anxiety, prioritizing specific phobias. The selected anxiety domains are entered in the system and will serve as targets in the intervention. The order in which the domains are targeted is randomized within the system, which means not the trainer but the system decides which anxiety domain will be targeted with exposure and which domain with cognitive restructuring. This minimizes chances of (unwanted) manipulation of the trainer (e.g. always target specific phobias with exposure and always target generalized anxiety with cognitive restructuring).

The intervention starts off with an introduction through messages between the trainer and the participant, and then psycho-education (session 1). In accord with the inhibitory learning model, exposure (session 2 to 5, see below) takes place before cognitive restructuring (session 6 and 7, see below). Because we chose to follow the inhibitory model, we could not randomize the order of exposure or cognitive restructuring over the participants. We, therefore, chose to target two anxiety domains, one for the working mechanism *exposure* and one for the working mechanism *cognitive restructuring*, and to randomize the order in which the domains are targeted. The first anxiety domain is targeted during exposure, the second domain during cognitive restructuring. This way, the net effect of the working mechanism on the child’s anxiety can be tested most validly while following the inhibitory learning model. The intervention ends with relapse prevention and saying goodbye to the trainer (session 8). Every week, one session takes place, except when the child wants to take a break during holidays.

Exposure is set up in line with the inhibitory learning model (described in: [40]). To accomplish this, we made the expectancy of the child about the feared situation explicit and aimed at applying variability during the exposure sessions. During session 2, the child is asked to step in an imaginary excavator to uncover the child’s anticipated negative outcome, or the child’s expectancy, of the feared situation. Participants are also encouraged to share the worst thing that might happen when confronted with their anxiety domain. The child’s expectations about the feared situation are tested during session 3, 4 and 5. For every one of his/her five expectations, the child indicates on a VAS scale how certain he/she is that the expectation will come true. We chose five expectations, to enable variation of testing situations, while keeping the number of testing situations feasible for an online early intervention. The trainer decides in which order these expectations will be tested. In accord with inhibitory learning, the order of exposures is not hierarchical, but rather starts off with a difficult experiment, to provide the child with a strong learning opportunity. The trainer also guides the child in minimizing the use of safety behavior and safety signals. During session 3, the child watches videos of other children preparing, performing and evaluating an exposure session based on inhibitory learning. Thereafter, the child chooses five rewards (e.g. getting to choose dinner, going to the cinema with mum/dad), one for every expectation he/she will test. Next, the child makes a first tracking plan, in which the child plans an experiment in which he/she will test the first expectation. In the tracking plan, the child describes how the expectation will be tested, where the expectation will be tested and who will assist the child. The child indicates on a calendar when this experiment will take place. Then, the child actually performs the experiment and shares the experience with the trainer. This can be done by uploading a movie clip or by describing the experiment in text or audio. After the exposure session, the child reflects on what happened during the exposure and on the possible non-occurrence of the anticipated negative outcome in the online workbook. The child reflects on the aspects of the expectation that were not correct, and completes another VAS scale indicating how certain the child is about the expectation after the experiment. The evaluation enhances the consolidation of the new learned association (CS – no US). During session 4 and 5, the child will design and complete the other four tracking plans.

Cognitive restructuring takes place during session 6 and 7, focusing on the second feared situation. During session 6, the child learns to detect dysfunctional thoughts and to replace dysfunctional thoughts by functional thoughts. In session 7, the child learns to use schemes that unravel anxious events into the four elements: neutral situation, anxious thought, anxious feeling, anxious behavior. The child gets acquainted with changing the dysfunctional, anxious thought within these schemes to a helping functional though, thereby positively affecting the child’s subsequent feelings and behaviors.

### Measurements

The measurement tools include questionnaires, an online task and an interview. The questionnaires and the online task are embedded in the ‘Learn to Dare!’ platform, and data are automatically entered into this platform. The diagnostic interview is performed by telephone by trained bachelors in psychology, who enter the data in a separate section within the platform. The diagnostic interviewers were never involved in providing the online CBT.

#### Child anxiety

##### Youth anxiety measure for DSM-5 (YAM-5, [54, 55])

The YAM-5 is a questionnaire that can be used to assess anxiety symptoms (or the anxiety level) in children and adolescents. This study focused on the child self-report version. The YAM-5 consists of two parts. Part I (28 items) taps symptoms of the major DSM-5 anxiety disorders, and thus contains the following subscales: separation anxiety disorder (6 items, e.g., I am afraid to go anywhere without my parents), selective mutism (4 items, e.g., At school, I don’t speak to the teacher at all), social anxiety disorder (6 items, e.g., I find it scary to meet new people), panic disorder (6 items, e.g., I panic for no reason), and generalized anxiety disorder (6 items, e.g., I worry about a lot of things). Part II (22 items) also contains 5 subscales covering the phobia types: animal (5 items, e.g., I am afraid of wasps), natural environment (4 items, e.g., I am afraid of the dark), blood-injection-injury (3 items, e.g., I am afraid of undergoing a small medical operation), other (4 items, e.g., I am afraid of people who are dressed up in costumes), and situational, which, in terms of fear content resembles agoraphobia (6 items, e.g., I am afraid when crossing a large town square). All items are rated on a 4-point Likert scale, ranging from 0 (never) to 3 (always). Good internal consistencies were demonstrated for the subscales of Part I and for the total scale of Part I and Part II. Furthermore, good test-retest reliability, good concurrent validity and good construct validity of both parts of the questionnaire were found.

##### Structured clinical interview for DSM-5 disorders for children (SCID, [56])

The SCID-junior is a structured clinical interview to assess the most common DSM-5 disorders in childhood, including all anxiety disorders. It is a DSM-5 modification of the Kid-SCID [57], a widely used interview for assessing DSM-IV childhood mental disorders that has moderate to good interrater reliability and internal consistency [58, 59]. The DSM-5 criteria for each disorder can be checked by a clinician, separately with the child, the parent(s), and other informants such as teachers, after which the clinician makes a “best judgment”.

#### Dysfunctional cognitions

##### Children’s automatic thoughts scale-negative/positive (CATS-N/P)

The CATS-N/P [60] is an extension of the CATS [61] and assesses negative (or: dysfunctional cognitions, DL’s) as well as positive thoughts in children. All of the items are scored on a 5-point scale, ranging from 0 (not at all) to 4 (all the time). The CATS contained the scales Physical Threat, Social Threat, and Personal Failure that measured negative thoughts. These subscales contain 30 items in total that can be added to form a sumscore, where higher scores reflect more negative thoughts. Positive thoughts are measured with the subscale Positive Thoughts that includes 10 items. These 10 items can also be added to form a subscore, with higher scores reflecting more positive thoughts. Hoogendoorn (2010) revealed good psychometric properties of the CATS-N/P, with total Cronbach’s alpha ranging between .90 and .96 for the total score and between .84 and .93 for the Positive Thoughts subscale.

#### Avoidance

##### Child avoidance measure self-report (CAMS, [62])

The CAMS contains eight items measuring a child’s tendency to avoid stimuli that elicit anxiety, fear, or worry (or: avoidance behavior, AB). The questionnaire presents a stem statement (When I feel scared or worried about something …) and the items covering various approaches to avoiding anxiety provoking stimuli, through passive and active avoidance, as well as refusal, (e.g., I try not to go near it). Items are rated on a Likert-type scale from Almost Never to Almost Always). The CAMS has good internal and test-retest reliability, concurrent and criterion validity, and treatment sensitivity. For the current study, the CAMS was translated to Dutch via a translate and back-translate procedure. One of the subgoals of this study is to psychometrically validate the Dutch version of the CAMS.

#### Child’s level of abstract reasoning

We measure the level of the children’s abstract reasoning skills to get an indication of the level of cognitive level (CL). We developed the abstract reasoning task ourselves to enable implementation in our online platform and enable a non-invasive adaptation specifically for our age group. Our abstract reasoning task is based on other freely available online abstract reasoning tasks and on the abstract reasoning task of the Matrix Reasoning of the fifth version Wechsler Intelligence Scale for Children (WISC-V, [63]). There are 10 tasks in which the child sees an array of abstract forms with one missing form. The child then has to select the correct form out of four alternatives.

### Analyses

Both the Statistical Packages for Social Sciences (SPSS, version 24) and R (R. C. Team, 2013) will be employed for performing the analyses. Multilevel analysis [64] is used to answer the various sub-questions. The study design is a randomized two-group factorial design with repeated measures of the dependent variables. Intervention versus control group makes the between subjects factor and time is the only within subjects factor. Alternatively, the design can be seen as a randomized two-group multilevel design with repeated measurements nested within subjects. The sub-questions correspond with different parameters to be estimated in the statistical model. The model can be seen as a moderated mediation [65] model in which the effect of the training is on the one hand by reducing dysfunctional cognitions and on the other hand by reducing avoidance. In addition, the path depends on the level of development of the child by reducing dysfunctional cognitions (see Fig. 1).

To test the primary research question, we will use regression model with the outcome Anxiety Level (AL) as dependent variable and condition (LTD vs WL) as predictor. Sex and Age will be added as extra predictors, plus interactions between predictors to see if the effects differ per sex and age category (LC). Model: AL = b0 + b1*Group + b2*Sex + b3*LC + b4*Group*Sex + b5*Condition*LC. To test the moderation and mediation models, we will use multilevel analysis [64]. The five assessments (T1,T2,T3,T4,T5) will be the lowest level, the children the second level. This model can be considered a moderated mediation model (Hayes, 2013). The mediation analysis will consist of 2 steps. 1st: Dysfunctional Cognitions (DC) and Avoidance Behavior (AB) will be dependent variables, in separate analyses. 2nd: Anxiety level (AL) will be the dependent variable. Time will be added and modelled as T = 0,1,2,3,4, to tested the linear increase of Anxiety Level (AL) during the intervention. We will use a random intercept model, because we expect individual differences to exist in the levels of DC, AB, AL. Step 1. DC = b0 + b1*Group + b2*Time VG = b0 + b1*Group + b2*Time. Step2. AL = b0 + b1*Group + b2*VG + b3*DC. To test the third hypothesis (moderation by cognitive level), we will perform a multilevel analysis as above. 1st: Dysfunctional Cognitions (DC) and Avoidance Behavior (AB) will be dependent variables, in separate analyses. Cognitive Level (CL) will be added as predictor in a hierarchical linear multilevel model, including the interaction with condition to test for the moderating effect of CL on the relation between condition and mediators. Time will be added and modelled as T = 0,1,2,3,4, to tested the linear increase of DC and AB during the intervention. We will use a random intercept model, because we expect individual differences to exist in the levels of DC and AB. DC = b0 + b1*Group + b2*CL + b3*Group*CL + b4*Time VG = b0 + b1*Group + b2*CL + b3*Group*CL + b4*Time. The variable Cognitive Level (CL) will be centered at zero. For the follow-ups, when interaction effects appear to be significant, simple slopes plots will be made to illustrate the interaction effect. The 95% confidence interval of the parameter estimates of the multilevel and logistic regression analyses will be used to assess the statistical and practical significance of the estimates. The standardized parameter estimates will be used as estimated effect sizes. For all interval variables univariate outlier detection will be applied by using the Inter Quartile Range (IQR): scores outside 1.5*IQR will be examined, in order to see whether these are genuine scores, and cannot be attributed to coding or other errors. If they are genuine the scores remain in the analysis, otherwise they are removed from the analysis. Since we will use multilevel analyses drop-outs can be kept in the analysis. Subjects with at least one wave (time point) with complete data will be used in the analysis. No imputation strategy will be applied. We will explore the time factor in more detail. Other coding schemes than a simple linear coding will be tested to see whether effect gradually weaken in time or take effect after a certain period. Furthermore, we will explore whether the proposed model has predictive value for the binary outcome (anxiety diagnosis).

## Discussion

Offering easy accessible interventions and providing insight into the working mechanisms of Cognitive Behavioral Therapy will contribute to optimizing Cognitive Behavioral Therapy for anxious youth.

## Data Availability

A detailed study design and analysis plan were preregistered at https://osf.io/g2avh, supporting data can be accessed via this register in the Open Science Framework.

## Abbreviations

AB: Avoidance Behavior
AL: Anxiety level
CATS-N/P: Children’s Automatic Thoughts Scale – Negative/Positive
CBT: Cognitive Behavioral Therapy
CL: Cognitive Level
CMS: Content Management System
CR: Conditional Reaction
CS: Conditional Stimulus
DC: Dysfunctional Cognition
DSM: Diagnostic and Statistical Manual of Mental Disorders
IQR: Inter Quartile Range
LAMP: Linux Apache MysqlPHP
LTD: Learn to Dare
RCT: Randomized Controlled Trial
SCID: Structured Clinical Interview for DSM-5 Disorders for children
US: Unconditional Stimulus
WL: Waitlist
YAM-5: Youth Anxiety Measure for DSM-5
